# Effects of the First Phase of Cardiac Rehabilitation Training on Self-Efficacy among Patients Undergoing Coronary Artery Bypass Graft Surgery

**Published:** 2018-07

**Authors:** Seyed Reza Borzou, Sasan Amiri, Mohsen Salavati, Ali Reza Soltanian, Gholamreza Safarpoor

**Affiliations:** 1 *Chronic* *Diseases (Home Care) Research Center, Faculty of Nursing* *and* *Midwifery,* *Hamadan University* *of Medical Sciences,* *Hamadan, Iran.*; 2 *Faculty of Nursing and Midwifery, Hamadan University of Medical Sciences, Hamadan, Iran.*; 3 *Department of Nursing, Faculty Nursing & Midwifery, Hamadan University of Medical Sciences, Hamadan, Iran.*; 4 *Modeling of Noncommunicable Diseases Research Center, School of Public Health, Hamadan University of Medical Sciences, Hamadan, Iran.*; 5 *Department of Cardiac Surgery, Hamadan University of Medical Sciences, Hamadan, Iran.*

**Keywords:** *Cardiac rehabilitation*, *Self efficacy*, *Education*

## Abstract

**Background:** Heart surgery is vital for patients with coronary artery diseases that do not respond to drug treatments. We aimed to determine the effects of the implementation of the first phase of a cardiac rehabilitation program on self-efficacy in patients after coronary artery bypass graft surgery (CABG).

**Methods:** This clinical trial study was conducted on 60 post-CABG patients by convenience sampling method in 2016. Those selected were randomly assigned to intervention (n=30) and control group (n=30). Overall, 72 hours after CABG, the first phase of the cardiac rehabilitation program both in theory and in practice (face-to-face and group methods) was conducted. Data were collected using a self-efficacy questionnaire completed by the patients in 3 stages: before the intervention, at discharge, and at 1 month after discharge. Data was analyzed by using analysis of covariance and repeated measures.

**Results:** The mean of age in the intervention and control groups was 61.60±11.72 and 57.97±13. 4 years, respectively. There were 16 (53.3%) male patients in each group. The mean score of self-efficacy was not significantly different between the 2 groups before the intervention (P=0.076), whereas it had a meaningful statistical difference between the 2 groups at discharge and 1 month afterward (P<0.001).

**Conclusion:** The implementation of the first phase of the cardiac rehabilitation program not only augmented self-efficacy in regard to independent daily activities but also lessened the need for the second phase of the program among our post-CABG patients.

## Introduction

Cardiovascular diseases are predicted to be the principal cause of disability in the world by the year 2020. These diseases are accountable for one-tenth of deaths in people aged less than 35 years, one-third of mortalities in individuals between 35 and 45 years old, and three-quarters of deaths in those over 45 years of age.^1^^-^^3^ Coronary artery diseases that cannot be treated with medical treatments require coronary artery bypass graft surgery (CABG).^4^^, ^^5^ This surgical modality is a common treatment for patients with coronary artery obstruction, under which category fall thousands of people annually. Iran boasts considerable experience with regard to cardiac surgery patients.^   6 ^ Heart surgery is associated with several complications, which, allied to old age and a history of cardiovascular diseases, impede daily activities in patients.^7^^, ^^8^ What exacerbates the post-surgical status of patients with heart trouble is a dearth of relevant information available to them on how to prevent such complications,^        9 ^ which underscores the significance of patient education as regards how to boost health status, prioritize prevention over treatment, and spend the convalescence period at home.^   10^

Needless to say, it is vitally important that post-CABG complications be reduced and cardiac output be increased in patients undergoing this surgical modality.^   11 ^ Cardiac rehabilitation programs could prevent readmission and reduce health-care costs.^12^^, ^^13^ These programs include a series of activities aimed at providing optimal physical, mental, and social conditions for surgical patients while slowing down the progression of their disease. Also needed are programs incorporating education about the modification of heart disease risk factors, behavioral changes, and psychological support. The goals of cardiac rehabilitation training are movement, independence, good mental performance, avoiding stress, maintaining a healthy social compatibility, and regaining abilities.^7^^, ^^13^ A swifter recovery of cardiac surgery patients is correlated with an increase in their exercise capacity; these patients should, accordingly, be encouraged to undergo cardiac rehabilitation after hospital discharge.^   14 ^ The first phase of a cardiac rehabilitation program seeks to augment physical capacity, but unfortunately few hospitals complete this phase.^   15 ^ In nonemergency CABG patients, the first stage involves moderate-to-severe walking with a view to enhancing exercise capacity and reducing the risk of atrial fibrillation.^5^^, ^^16^ Such activities are associated with higher self-efficacy and, thus, higher self-confidence for the continuation of the rehabilitation program among these patients.^5^^,^^   17 ^ Higher feelings of self-efficacy nurture the belief in patients that they can experience positive changes.^   18 ^ Indeed, for patients participating in rehabilitation programs, self-efficacy is a significant predictor of behavioral changes.^        19 ^ It is worthy of note, however, that the first phase of cardiac rehabilitation programs is only a stage toward returning patients to their regular daily activities and the fulfillment of the next stages is required to fully address patients’ medications, diets, and mental status.

The present study aimed to determine the effects of the implementation of the first phase of a cardiac rehabilitation program on self-efficacy in post-CABG patients. 

## Methods

The present 2-phase clinical randomized trial was performed in Farshchian Heart Hospital, in the western Iranian city of Hamadan, in 2016, on 60 post-CABG patients. With a type I error (α=0.05%) and a power test of about 90%, at least 26 patients were required in each group. Nonetheless, in order to avoid bias resulting from attrition, we recruited 30 patients for each group. 

This study was registered as a clinical trial in the Iranian Registry of Clinical Trials (IRCT2016013126294N1) and was approved by the Ethics Committee of Hamadan University of Medical Sciences (IR.UMSHA.REC.1394.430). 

The independent variable was the cardiac rehabilitation program, and self-efficacy was considered the dependent variable. The sample comprised post-CABG patients admitted to the intensive care unit (ICU) 72 hours postoperatively. The inclusion criteria consisted of age between 30 and 75 years, full awareness of time and space, ability to understand and speak the Farsi language, minimum ability to read and write, having no history of heart surgery, having no motion disorders, and ejection fraction greater than 30%. The exclusion criteria were comprised of uncontrolled arrhythmias, severe and persistent chest pain, and refusal to assist in data collection. 

The sampling was conducted in 2 stages. First, one group was selected randomly so that the selection was not affected by the education level. Then, the randomized sampling method was used. 

Written informed consent was obtained from all the patients, who were assured that their information would remain confidential. 

In the intervention group, the first phase of the cardiac rehabilitation program was performed in 3 sessions. This program was started 72 hours after surgery and continued until discharge time. The control group received the routine care and therapy. The data collection instrument was composed of 2 parts. The first part covered variables such as age, sex, height, weight, body mass index, place of residence, occupation, supplementary insurance, marital status, education level, history of hospitalization, history of illness, history of smoking, on/off pump surgery, number of grafts, ejection fraction, and food and drug allergy. The second part contained a cardiac-efficacy questionnaire, which included a self-efficacy questionnaire, adapted from a myocardial infarction self-efficacy questionnaire. The cardiac-efficacy questionnaire had 3 dimensions, namely general self-efficacy, exercise self-efficacy, and feeling of self-efficiency, and was composed of 32 questions carrying a total score of 160. Questions 1 to 10 were on general self-efficacy, 11 to 20 on exercise self-efficacy, and 21 to 32 on feeling of self-efficiency. Questions 1 to 20 had 100 points, and questions 21 to 32 had 60 points. The questions were ranked on a 5-point Likert Scale: always, often, sometimes, rarely, and never.

The validity and reliability of the questionnaire after making adjustments for the demographic data based on the inclusion criteria were determined via content validity tools by 8 nursing faculty members and 3 cardiology and cardiac surgery faculty members, who confirmed a Cronbach’s alpha reliability coefficient of 0.76 and a correlation coefficient of 0.74.^   20 ^

The demographic and cardiac-efficacy-related questions were completed in 3 stages: during hospital stay (in the intervention group before the intervention), at discharge, and at 1 month after discharge. Patients were selected and assessed after the stabilization of their condition. 

Seventy-two hours after the patients were transferred to the ICU, the first session was commenced with a view to determining the number of sessions and duration of each session. 

In this phase, the program comprised: (1) theoretical training about heart anatomy and disease, acute coronary syndrome, heart disease symptoms, and adjustment of modifiable risk factors and (2) implementation of the exercises. The exercises were comprised of deep breathing and active movements of the limbs while lying on the back, exercising in a semi-sitting position in bed, exercising in a sitting position next to bed, and repeating the previous exercises while sitting on a chair. Every stage was done at least twice a day, each time for 10 to 15 minutes. Booklets and pamphlets were given to the patients in preparation for the next session. 

In the second session, held on the day following the first session, the patients were provided with necessary explanations based on their scores on the theoretical and practical questions so that they could score the maximum point. The explanations covered food and drug regimens, chest pain control, pulse control, and how to warm up and cool down before and after the physical exercise. Thereafter, the patients were asked to perform exercises in the standing position (e.g., trunk rotation and rotational movements of the scapula and arm), to walk unaccompanied, to bend and straighten the knees, to twist the body in different directions, and to repeat the previous moves. 

The third session was held on the day after the second session and before discharge. All the previous activities were reviewed and appropriate activities were scheduled from discharge to readmission to the clinic. In the second and third sessions, the group method was applied in the patients’ rooms. After discharge, the patients were followed up by telephone to monitor their mental health twice a week.

For the statistical analyses, the statistical software SPSS for Windows, version 16.0 (Chicago, SPSS Inc.), was used. Repeated measures analysis was used to compare the self-efficacy dimensions at the 3 study time points in the intervention and control groups. Indicators such as frequency distribution were used to describe the data collected. 

## Results

The mean±standard deviation of age was 61.60±11.72 years in the intervention group and 57.97±13. 4 years in the control group. The number of male and female patients was equal in the 2 groups: 16 (53.3%) men and 14 (46.7%) women. The average body mass index was 26.03±4.49 kg/m^2^ in the intervention group and 26.00±4.59 kg/m^2^ in the control group. Paired *t*-tests were used to check the normality of age and the body mass index, and the Fisher exact test was applied for the patients’ sex.

After the homogenization of the variables of occupation, marital status, education level, number of grafts, surgery on and off the pump, ejection fraction, and history of illness, the 2 study groups were compared using the Fisher exact test and the Pearson test (Table 1).

The homogenization of the efficacy dimensions was evaluated using the Kolmogorov-Smirnov test. The results of the repeated analysis of variance for factor and group effects showed a significant difference between the 2 groups at the 3 study time points in terms of the mean score of general self-efficacy (P<0.001) and exercise self-efficacy (P<0.001). The mean score of the feeling of self-efficacy before intervention was statistically significantly different between the 2 groups (P=0.015). The mean score of the feeling of self-efficacy was adjusted in the intervention and control groups before intervention using the analysis of covariance: there were significant differences between the intervention and control groups at discharge and 1 month afterward (P<0.001). Finally, there were significant statistical differences for factor and group effects in the total score of self-efficacy between the intervention and control groups (P<0.001) (Table 2). 

The results are expressed as numbers and percentages for 30 patients in the intervention group and 30 patients in the control group. The statistical analysis (repeated analysis of variance) for factor and group effects showed significant differences in the total score of self-efficacy before the intervention, at discharge, and at 1 month after discharge between the intervention and control groups (P*<*0.001).

**Table 1:** Distribution of the clinical and demographic characteristics in the patients undergoing coronary artery bypass grafting

	Intervention Group (n=30)	Control Group (n=30)	P
Age (y)	61.6±11.7	57.97±13.1	0.262
			
Sex			1
Male	16 (53.3)	16 (53.3)	
Female	14 (46.7)	14 (46.7)	
			
Occupation			0.651
Worker	3 (10.0)	1 (3.3)	
Clerk	8 (26.7)	11 (36.7)	
Housewife	13 (43.3)	16 (53.3)	
Self-employed	3 (20.0)	2 (6.7)	
Marital Status			0.704
Single	1 (3.3)	2 (6.7)	
Married	28 (93.3)	26 (86.7)	
Divorced	1 (3.3)	1 (3.3)	
Widow	0	1 (3.3)	
Education			0.284
Primary school	19 (63.3)	16 (53.3)	
Below high school diploma	5 (16.7)	8 (26.7)	
High school diploma	2 (6.7)	5 (16.7)	
University qualifications	4 (13.3)	1 (3.3)	
Number of Grafts			0.688
Two grafts	3 (10.0)	4 (13.3)	
Three grafts	27 (90.0)	26 (86.7)	
Heart Surgery			0.280
On pump	23 (76.7)	20 (66.6)	
Off pump	7 (23.3)	10 (33.4)	
Ejection Fraction (%)			0.855
30–39	4 (13.3)	2 (6.7)	
40–49	18 (60.0)	19 (63.3)	
≥50	8 (26.7)	9 (30.0)	
			0.304
Hypertension	6 (20.0)	10 (33.3)	0.343
Hyperlipidemia	4 (13.3)	5 (16.7)	0.500
Diabetes mellitus	5 (16.7)	1 (3.3)	0.195
Cardiovascular disease	9 (30.0)	11 (36.7)	0.584

* The results are expressed as mean±SD or n (%).

**Table 2:** Comparison of the different dimensions of self-efficacy in the patients undergoing CABG

	Intervention Group (SD) repeated analysis of variance	Control Group (SD) repeated analysis of variance	P independent *t*-test
General self-efficacy			
Before intervention	36.57 (1.09)	35.73 (1.09)	0.590
At discharge	41.43 (0.84)	36.03 (0.84)	<0.001
One month after discharge	42.23 (1.04)	36.30 (1.04)	<0.001
	F=11.25 P<0.001	F=7.95 p<0.001	
			
Exercise Self-efficacy			
Before intervention	28.13 (1.56)	31.3 (1.59)	0.136
At discharge	43.03 (1.42)	30.90 (1.44)	<0.001
One month after discharge	44.47 (1.35)	30.83 (1.37)	<0.001
	F=40.85 P<0.001	F=45.40 P<0.001	
			
Feeling of self-efficacy			
Before intervention	39.87 (1.27)	44.37 (1.27)	0.015
At discharge	47.83 (1.07)	43.27 (1.93)	<0.004
One month	50.50 (1.19)	44.07 (1.56)	<0.001
after discharge	F=19.62 P<0.001	F=26.17 P<0.001	
			
Total self-efficacy			
Before intervention	104.57 (14.50)	111.63 (15.81)	0.076
At discharge	132.30 (10.25)	110.37 (13.34)	<0.001
One month after discharge	137.20 (14.75)	111.70 (13.39)	<0.001
	F=63.46 P<0.001	F=67.94 P<0.001	

SD, Standard deviation; CABG, Coronary artery bypass graft

## Discussion

About 80% of patients with coronary artery disease undergo CABG, and every single one of them needs to undertake the first phase of the cardiac rehabilitation program. Self-efficacy makes a great deal of difference in how an individual feels, thinks, and acts; it can increase the individual’s motivation to take action. Indeed, those who have higher self-efficacy are capable of taking on and fulfilling more challenging tasks. 

The results of the present study demonstrated that the first stage of the cardiac rehabilitation program, composed of theoretical and practical sessions, boosted self-efficacy in daily activities among our post-CABG patients. At the time of discharge and 1 month later, the self-efficacy scores in all the dimension of self-efficacy were significantly different between our intervention and control groups. Our results were concordant with those in a study on the effects of phase I cardiac rehabilitation on anxiety insofar as the intervention and control groups had no difference in terms of the anxiety scores before the intervention; nonetheless, the intervention led to a significant difference between the 2 groups (P<0.001).^   20 ^ The mean±standard deviation of the self-efficacy scores of the patients in the experimental group exhibited a significant difference before and after the intervention. Vibulchai et al.^   21 ^ reported that cardiac rehabilitation via self-massage, exercise, and walking in three 40-minute sessions improved self-efficacy 1 month after the intervention among patients with myocardial infarction (P<0.001). Elsewhere, Wang et al.^5^ devised a regular multi-exercise training plan and reported improvement in the 6-min walk test, heart rate, and self-efficacy in their study patients at discharge and 1 month thereafter. There is concordance between the findings of that study and ours insofar as our study patients maintained their acquired self-efficacy at 1 month after discharge as well. The application of the cardiac rehabilitation program in another study with a focus on psychological factors and quality of life in patients with coronary artery disease boosted their self-efficacy, self-regulation, and self-care.^   22^

The role of self-efficacy and motivation in predicting short-term and long-term adherence to physical activity among patients with congenital heart diseases was the objective of another previous study by D'Angelo et al.,^        23 ^ who showed that motivation and self-efficacy were effective in following sports activities. In the short term (6 mon), the role of self-efficacy and in the long term (12 mon), the role of motivation were effective in adherence to exercise programs (P<0.001). The results of that study chime in with ours inasmuch as in terms of adherence to the health-care team’s recommendations in the short term (1–6 mon), the role of self-efficacy was more prominent among our patients.

Zhou et al.^   24 ^ reported that the differences between the self-efficacy scores before and after the intervention (6 mon), as well as at 12 month after the intervention, were statistically significant (P*<*0.05) in their study patients. Poortaghi et al.^   25 ^ carried out a home-based rehabilitation program for patients with heart disease.^   25^

## Conclusion

The implementation of the first phase of the cardiac rehabilitation program both increased self-efficacy with respect to independent daily activities and reduced the need for the second phase of the program among our post-CABG patients. 

## Notes:

***This paper should be cited as:*** Borzou SR, Amiri S, Salavati M, Soltanian AR, Safarpoor G. Effects of the First Phase of Cardiac Rehabilitation Training on Self-Efficacy among Patients Undergoing Coronary Artery Bypass Graft Surgery. J Teh Univ Heart Ctr 2018;13(3):126-131.
